# The complete chloroplast genome sequence of *Citrus sinensis *(L.) Osbeck var 'Ridge Pineapple': organization and phylogenetic relationships to other angiosperms

**DOI:** 10.1186/1471-2229-6-21

**Published:** 2006-09-30

**Authors:** Michael G Bausher, Nameirakpam D Singh, Seung-Bum Lee, Robert K Jansen, Henry Daniell

**Affiliations:** 1 USDA-ARS, Horticultural Research Laboratory, Fort Pierce, FL 34945–3030, USA; 2 Dept. of Molecular Biology & Microbiology, University of Central Florida, Biomolecular Science, Building #20, Orlando, FL 32816–2364, USA; 3 Section of Integrative Biology and Institute of Cellular and Molecular Biology, Patterson Laboratories 141, University of Texas, Austin, TX 78712, USA

## Abstract

**Background:**

The production of *Citrus*, the largest fruit crop of international economic value, has recently been imperiled due to the introduction of the bacterial disease *Citrus *canker. No significant improvements have been made to combat this disease by plant breeding and nuclear transgenic approaches. Chloroplast genetic engineering has a number of advantages over nuclear transformation; it not only increases transgene expression but also facilitates transgene containment, which is one of the major impediments for development of transgenic trees. We have sequenced the *Citrus *chloroplast genome to facilitate genetic improvement of this crop and to assess phylogenetic relationships among major lineages of angiosperms.

**Results:**

The complete chloroplast genome sequence of *Citrus sinensis *is 160,129 bp in length, and contains 133 genes (89 protein-coding, 4 rRNAs and 30 distinct tRNAs). Genome organization is very similar to the inferred ancestral angiosperm chloroplast genome. However, in *Citrus *the *infA *gene is absent. The inverted repeat region has expanded to duplicate *rps19 *and the first 84 amino acids of *rpl22*. The *rpl22 *gene in the IRb region has a nonsense mutation resulting in 9 stop codons. This was confirmed by PCR amplification and sequencing using primers that flank the IR/LSC boundaries. Repeat analysis identified 29 direct and inverted repeats 30 bp or longer with a sequence identity ≥ 90%. Comparison of protein-coding sequences with expressed sequence tags revealed six putative RNA edits, five of which resulted in non-synonymous modifications in *petL*, *psbH*, *ycf2 *and *ndhA*. Phylogenetic analyses using maximum parsimony (MP) and maximum likelihood (ML) methods of a dataset composed of 61 protein-coding genes for 30 taxa provide strong support for the monophyly of several major clades of angiosperms, including monocots, eudicots, rosids and asterids. The MP and ML trees are incongruent in three areas: the position of *Amborella *and Nymphaeales, relationship of the magnoliid genus *Calycanthus*, and the monophyly of the eurosid I clade. Both MP and ML trees provide strong support for the monophyly of eurosids II and for the placement of *Citrus *(Sapindales) sister to a clade including the Malvales/Brassicales.

**Conclusion:**

This is the first complete chloroplast genome sequence for a member of the Rutaceae and Sapindales. Expansion of the inverted repeat region to include *rps19 *and part of *rpl22 *and presence of two truncated copies of *rpl22 *is unusual among sequenced chloroplast genomes. Availability of a complete *Citrus *chloroplast genome sequence provides valuable information on intergenic spacer regions and endogenous regulatory sequences for chloroplast genetic engineering. Phylogenetic analyses resolve relationships among several major clades of angiosperms and provide strong support for the monophyly of the eurosid II clade and the position of the Sapindales sister to the Brassicales/Malvales.

## Background

Chloroplasts are dynamic organelles of prokaryotic origin within the plant cell that house the photosynthetic apparatus. In addition to photosynthesis, other important metabolic activities take place within chloroplasts including the production of starch, certain amino acids and lipids, some of the colorful pigments in flowers, vitamins and several key aspects of sulfur and nitrogen metabolism. Chloroplasts possess their own genome and a full complement of transcriptional and translation machinery to express their genetic information. In particular, chloroplast gene expression machinery is a distinctive assembly of prokaryotic, eukaryotic, and phage-like components–likely the result of acquisition of a great number of regulatory proteins during evolution. The presence of nucleic acids within chloroplasts was established in 1963 [1]. This subsequently led to the selection of cpDNA as one of the first candidates for complete genome sequencing [2]. Studies of the organization and evolution of chloroplast genomes have been rapidly expanding due to the availability of the number of completely sequenced genomes published in the past decade. Fifty-four completed genomes are available from various land plant lineages, with the best representation (36 species) from flowering plants. Comparative studies indicate that chloroplast genomes of land plants are highly conserved in both gene order and gene content [3]. Moreover, the substitution rate in cpDNA is much lower than in nuclear DNA and significantly reduced in the inverted repeat regions as compared to the single copy regions [4].

Chloroplast bioengineering offers a number of advantages over nuclear transformation including high levels of transgene expression and gene containment [5]. In addition, chloroplast genetic engineering has also become a powerful tool for basic research in biogenesis and function of this organelle. This approach has helped unveil a wealth of information about cpDNA replication origins, introns, maturases, translation elements, proteolysis, import of proteins and several other processes [5]. However, this technology is readily feasible only in tobacco. Lack of complete chloroplast genome sequence is still one of the major limitations preventing the expansion of chloroplast bioengineering to other useful crops. Transgene integration into the chloroplast genome occurs exclusively by homologous recombination of chloroplast DNA flanking sequences. Therefore, chloroplast genome sequence analysis is crucial for identification of spacer regions to integrate transgenes at optimal positions as well as the identification of endogenous regulatory sequences that support optimal expression of transgenes [5]. Prior to 2004 only seven published crop chloroplast genomes were available and this number has increased to 23 during the past two years [6]. Furthermore, the availability of genome sequence information has also made it possible to study evolutionary relationships among chloroplast and nuclear genomes [7].

*Citrus *is the largest fruit crop of international economic value because of its many uses including its value as a nutritive food source and for its valuable essential oils utilized by the food, pharmaceutical, and cosmetic industries. The valuable *Citrus *industry in Florida (USA) has recently been put in peril because of the accidental introduction of the exotic disease *Citrus *canker. This bacterial disease, which can infect all cultivars of *Citrus*, is the result of infection by *Xanthomonas pv citri *[8]. Elimination of this disease by eradication has resulted in a cost of $1.2 billion (US) and the destruction of 7 million commercial and 5 million nursery and residential trees (pers. comm. T.R. Gottwald). Attempts at resistance breeding in *Citrus *are impeded by many biological characteristics, such as juvenility, incompatibility, heterozygosity, a narrow genetic basis, and nucellar embryony. In this context, genetic engineering of the chloroplast genome with non-host resistance traits would be an effective alternative for transferring desirable traits because of its many advantages over nuclear transformation [5]. However, for *Citrus*, genetic improvement through chloroplast transformation has been limited due to the lack of available chloroplast genome sequence, not only in the genus *Citrus *but also in the entire family Rutaceae.

In this article, we report on the complete sequence of the chloroplast genome of *Citrus sinensis *(L.) Osbeck var. 'Ridge Pineapple', which is the first published whole genome sequence of a member of the family Rutaceae and order Sapindales. We describe the organization of this genome and we present a phylogenetic analysis of *Citrus *and 27 other angiosperm chloroplast genomes based on 61 shared protein-coding genes. The phylogenetic comparisons enable an examination of relationships among several major clades of angiosperms.

## Results

### Size, gene content, order and organization of the *Citrus *chloroplast genome

**Figure 1:**
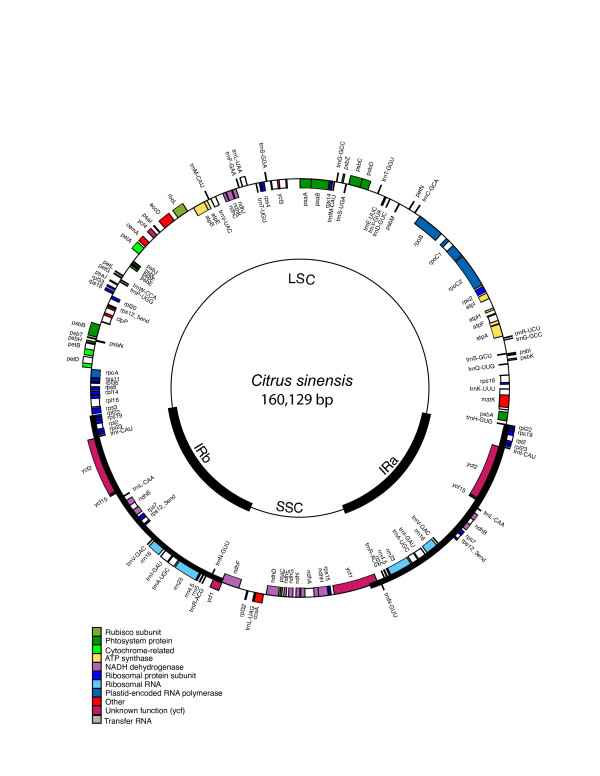
Circular gene map of *Citrus sinensis *chloroplast genome. The thick lines indicate the extent of the inverted repeats (IRa and IRb, 26,996 bp), which separate the genome into small (SSC, 18,393 bp) and large (LSC, 87,744) single copy regions. Genes on the outside of the map are transcribed in the clockwise direction and genes on the inside of the map are transcribed in the counterclockwise direction.

The complete nucleotide sequence of the chloroplast genome of *Citrus sinensis *(L.) Osbeck var 'Ridge Pineapple' has been determined (Fig. 1). This genome is 160,129 bp in length and includes a pair of inverted repeats (IR) of 26,996 bp separated by small and large single copy (SSC, LSC) regions of 18,393 bp and 87,744 bp, respectively. A total of 133 genes was detected, 113 are single copy, while 20 are duplicated in inverted repeat regions. Eighty-nine genes code for proteins, including nine genes duplicated in the inverted repeat. There are four rRNA genes and 30 distinct tRNAs, 7 of which are duplicated in the inverted repeat. Seventeen genes have introns, 14 of which contain a single intron while three (*clpP*, *rps12*, *ycf3*) have two introns. The genome consists of 49.94% protein-coding, 42.65% non-coding, 1.74% tRNA and 5.65% rRNA genes. The GC and AT content in the *Citrus *chloroplast genome is 38.48% and 61.52%, respectively. The overall AT content is similar to tobacco (62.2%), rice (61.1%) and maize (61.5%). The AT content of the LSC and SSC regions are 63.19% and 66.66% respectively, whereas that of the IR-regions is 57.05% due to the presence of an rRNA gene cluster. *infA*, a gene coding for a translation initiation factor in other plant species, is absent in the *Citrus *genome. The inverted repeat region has expanded to duplicate *rps19 *and the first 84 amino acids of *rpl22*. The *rpl22 *gene in the IRb region has a nonsense mutation resulting in 9 stop codons. Both the IR expansion and the presence of internal stop codons in *rpl22 *were confirmed by PCR amplification and sequencing using primers that flank the IR/LSC boundaries.

### Repeat analysis

Repeat analysis identified 29 direct and inverted repeats 30 bp or longer with a sequence identity ≥ 90% (Table 1). The longest repeat, other than the IR is 53 bp in length. Most of the repeated sequences are located in the intergenic regions while some are in protein-coding regions (i.e., *psaA*, *psaB*; Table 1).

### Variation between coding sequences and cDNAs

DNA and EST sequences were compared by aligning the ~92,000 publicly available *Citrus sinensis *expressed sequence tag (EST) sequences with the genes extracted from completed *Citrus *chloroplast genome sequence. Five non-synonymous nucleotide substitutions were identified in the protein-coding transcripts of *petL*, *psbH*, *ycf2 and ndhA *(Table 2). In *ycf2 *two amino acid substitutions were found, which resulted in a change from hydrophobic non-polar to hydrophilic acidic and hydrophilic polar amino acids, respectively. The substitution in the *ndhA *protein resulted in a change from a hydrophilic polar to a hydrophobic non-polar. In contrast, only one synonymous substitution was detected in transcripts coding for *rps18*. In non-protein-coding regions, seven additional differences were detected, including one in the intron of *ycf3 *and five in the ribosomal RNA gene *rrn23 *(Table 2). The differences could be due to mRNA editing, sequencing error, or polymorphisms between the tissues used for genome versus EST sequencing.

### Phylogenetic analysis

**Figure 2:**
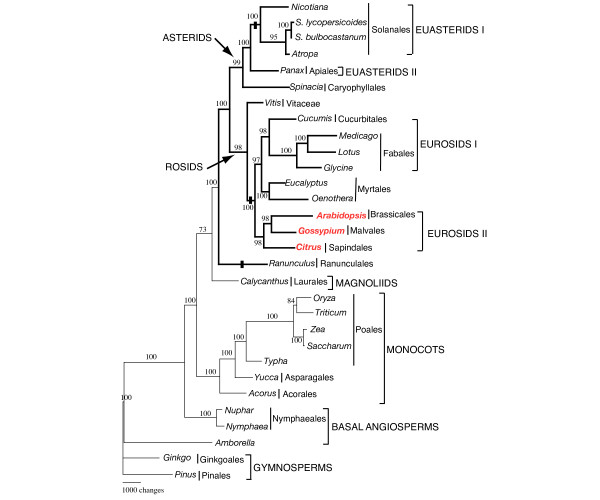
Maximum parsimony tree based on 61 chloroplast protein-coding genes [69]. The single most parsimonious phylogram has a length of 53,085, a consistency index of 0.45 (excluding uninformative characters), and a retention index of 0.60. Numbers at nodes indicate bootstrap support values and branch length scales are shown at base of the tree. Taxa in red are members of the eurosid II clade. Thicker lines in tree indicate members of eudicots. Black bars indicate lineages that have lost *infA*. Accession numbers for taxa are: *Pinus*, NC_001631; *Ginkgo*, DQ069337-DQ069702, *Amborella*, NC_005086, *Nuphar*, DQ069337-DQ069702, *Nymphaea*, NC_006050; *Acorus*, DQ069337-DQ069702; *Oryza*, NC_001320; *Saccharum*, NC_006084; *Triticum*, NC_002762; *Typha*, DQ069337-DQ069702; *Yucca*, DQ069337-DQ069702; *Zea*, NC_001666; *Calycanthus*, NC_004993; *Arabidopsis*, NC_000932; *Atropa*, NC_004561; *Cucumis*, NC_007144; *Eucalyptus*, AY780259; *Glycine*, NC_007942; *Gossypium*, NC_007944; *Citrus*, DQ864733; *Lotus*, NC_002694; *Medicago*, NC_003119; *Nicotiana*, NC_001879; *Oenothera*, NC_002693; *Panax*, NC_006290; *Ranunculus*, DQ069337-DQ069702; *Solanum lycopersicum*, DQ347959; *Solanum bulbocastanum *NC_007943; *Spinacia*, NC_002202; *Vitis*, NC_007957.

**Figure 3:**
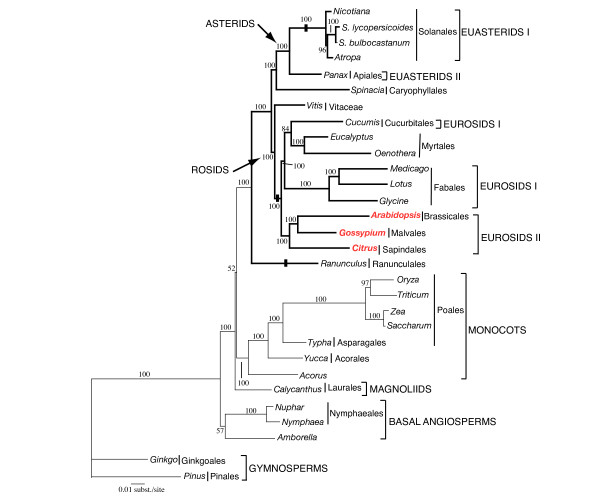
Maximum likelihood tree based on 61 chloroplast protein-coding genes. The single maximum likelihood phylogram has a ML value of – lnL = 305916.24523. Numbers at nodes indicate bootstrap support values and branch length scale is shown at base of the tree. Taxa in red are members of the eurosid II clade. Thicker lines in trees indicate members of eudicots. Black bars indicate lineages that have lost *infA*.

The data matrix for phylogenetic analyses included 61 protein-coding genes for 30 taxa, including 28 angiosperms and two gymnosperm outgroups (*Pinus *and *Ginkgo*). The data set comprised 45,573 aligned nucleotide positions but when the gaps were excluded there were 39,618 characters. Maximum Parsimony (MP) analyses resulted in a single, fully resolved tree with a length of 53,085, a consistency index of 0.45 (excluding uninformative characters) and a retention index of 0.60 (Fig. 2). Bootstrap analyses indicated that 25 of the 27 nodes were supported by values ≥ 95%. Maximum Likelihood (ML) analysis resulted in a single tree with a ML value of – lnL = 305916.24523 (Fig. 3). The ML and MP trees differed in the relationships among three groups (compare Figs. 2, 3). First, the MP tree placed *Amborella *alone as the earliest diverging angiosperm lineage and this position was strongly supported with a 100% bootstrap value. In contrast, the ML tree provided weak support (57% bootstrap value) for a sister relationship between *Amborella *and the Nymphaeales at the base of angiosperms. Second, in the MP tree *Calycanthus*, the only representative of magnoliids, was positioned sister to eudicots with moderate bootstrap support of 73%. In the ML tree, *Calycanthus *was weakly supported (52% bootstrap value) as sister to a clade that includes both monocots and eudicots. Third, the monophyly of the eurosid I clade was strongly supported in the MP tree (98% bootstrap value), whereas the ML tree does not support eurosid I monophyly. Both MP and ML analyses provided strong support for the monophyly of eurosid II and for the placement of *Citrus *(Sapindales) sister to a clade that includes *Gossypium *(Malvales) and *Arabidopsis *(Brassicales).

## Discussion

### Implications for integration of transgenes

Chloroplast genetic engineering offers several advantages, including a high-level of transgene expression [9], multi-gene engineering in a single transformation event [10], transgene containment via maternal inheritance [11] or cytoplasmic male sterility [12], lack of gene silencing [9,13], position effect due to site specific transgene integration [14], and lack of pleiotropic effects due to sub-cellular compartmentalization of transgene products [15-17]. Apart from expressing therapeutic agents [18], biopolymers [19] or transgenes to confer valuable agronomic traits, including herbicide resistance [20], disease resistance [21], insect resistance [22], drought tolerance [16], salt tolerance [23], and phytoremediation [24], chloroplast genetic engineering has been used to study chloroplast biogenesis and function, revealing the mechanisms of DNA replication origins, intron maturases, translation elements and proteolysis, import of proteins, and several other processes [25]. Despite the potential of chloroplast genetic engineering, this technology has only recently been extended to the major crops, including soybean [26], carrot [23], lettuce [27], and cotton [28].

The availability of complete sequences of chloroplast genomes enhances their use for genetic engineering. In chloroplast transformation, finding appropriate intergenic spacer regions is very important for efficient integration of transgenes. In tomato and potato, researchers have used *trnfM-trnG*, *rbcL*-*accD*, *trnV*-*3*'-*rps12*, and *16S rRNA-orf 70B *intergenic spacer regions of tobacco to integrate transgenes [29-31]. Unfortunately, none of these regions have 100% sequence identity [6]. For example, the intergenic spacer region between *rbcL *and *accD *of potato and tobacco shows only 94% sequence identity. Subsequently, potato chloroplast transformants are generated at 10–30 times lower frequencies than tobacco [31]. Similarly, the *trnfM *and *trnG *intergenic spacer region used for tomato chloroplast transformation has only 82% sequence identity with tobacco, resulting in inefficient transgene integration. There are major deletions in the tomato chloroplast genome in this intergenic spacer region when compared to tobacco, which was used for transformation [6]. Therefore, the development of species-specific vectors for transgene integration would enable the use of any of the intergenic spacer regions within the respective chloroplast genomes [6]. Moreover, genome organization is different among some species. For instance the *rbcL *and *accD *genes are adjacent in tobacco and most other angiosperm chloroplast genomes, including *Citrus*. However, they are not adjacent in the soybean chloroplast genome because an inversion has altered gene order [32]. These examples emphasize the importance of choosing appropriate intergenic spacer regions for chloroplast transformation.

### Genome organization

Gene order of the *Citrus *genome is identical to the published genome sequences of the *Solanaceae *[6], which have the inferred ancestral angiosperm genome organization [3]. The *rps19 *gene and the first 84 amino acids of *rpl22*, which generally are single copy in the LSC on the IRb side, have been duplicated in *Citrus*. Thus, there is a complete, second copy of *rps19 *and a truncated copy of *rpl22 *adjacent to *trnH*. This duplication is likely due to an expansion of IRb at the LSC junction, a common process in chloroplast genomes [33]. The gene content of *Citrus *is also very similar to most other angiosperm chloroplast genomes. However, *infA*, a gene coding for a translation initiation factor in other plant species, is absent in the *Citrus *genome, and *rpl22 *is apparently not functional due to a frame shift mutation. Millen et al. [34] demonstrated at least 24 independent losses of *infA *in angiosperms, and in four lineages this gene has been shown to be transferred to the nucleus. Three of these losses are evident in our phylogeny based on cpDNA sequences (indicated by bars in Figs. 2, 3). Among the rosid genomes sequenced the *infA *loss has occurred only once and this change supports the basal split between *Vitis *and the rest of the rosids (Figs. 2, 3). The *rpl22 *gene in the IRb region has a nonsense mutation resulting in 9 stop codons indicating that this gene is not functional. This was confirmed by PCR amplification and sequencing using primers that flank the IR/LSC boundaries. The *rpl22 *gene has been reported to be missing in legume chloroplast genomes and the import of nuclear encoded protein has been demonstrated [32,35]. Our group recently reported that *rpl22 *was also missing in the cotton chloroplast genome [36] but it turns out that this was an annotation error. The lack of a functional copy of *rpl22 *in *Citrus *should be investigated further, including an expanded sampling of members of the Rutaceae and Sapindales.

Repeat analysis identified 29 direct and inverted repeats 30 bp or longer with a sequence identity ≥ 90% in the *Citrus *chloroplast genome with the longest repeat, other than the IR, 53 bp in length (Table 1). The presence of dispersed repeats in chloroplast genomes, especially in intergenic spacer regions, has been reported in a number of angiosperm lineages, including other rosids [37].

### Phylogenetic implications

Phylogenies based on 61 protein-coding genes (Figs. 2, 3) generally agree with several recent studies based on multiple genes or complete chloroplast genomes [37-39]. Areas of congruence that are strongly supported include the monophyly of monocots and their sister relationship to eudicots, monophyly of rosids and asterids, and the sister relationship between Caryophyllales (represented by *Spinacia*) and asterids.

Our chloroplast genome trees (Figs. 2, 3) indicate that the earliest diverging angiosperm lineage is either *Amborella *or *Amborella *+ Nymphaeales. This incongruence between MP and ML trees was noted previously [37,39]. This same incongruence was observed in a multigene phylogeny that includes nine genes from the chloroplast, mitochondrial and nuclear genomes [40]. In this case, phylogenies for chloroplast genes supported the *Amborella *basal hypothesis, whereas mitochondrial genes supported *Amborella *+ Nymphaeales as the earliest angiosperm lineage.

A second incongruence between MP and ML trees concerns the position of the magnoliid *Calycanthus*, although bootstrap support for the different relationships is weak (Figs. 2, 3). The MP tree places *Calycanthus *sister to eudicots, whereas the ML tree positions this taxon sister to a clade that includes both monocots and eudicots. This same incongruence was observed in previous phylogenetic analyses based on the 61 protein-coding chloroplast genes [37,39]. The position of magnoliids continues to be controversial. Several molecular phylogenies have suggested different sets of relationships among magnoliids, monocots, and eudicots. Phylogenies based on phytochrome [41] and 17 chloroplast [42] genes placed magnoliids sister to monocots + eudicots but bootstrap support was weak. Several studies supported monocots as the sister group of magnoliids + eudicots [43-45] but bootstrap support was again weak. Both *matK *[46] and three gene [38] phylogenies suggested that eudicots are sister to magnoliids + monocots. Finally, the nine-gene phylogeny of Qiu et al. [40] recovered all three of these sets of relationships depending on the phylogenetic methods (MP or ML) and the genes used but support was very weak in each case. The different resolutions of relationships of magnoliids are greatly affected by taxon sampling and phylogenetic methodology. The affects of both of these phenomena have been discussed in several recent papers on the utility of whole chloroplast genomes for phylogenetic reconstruction of angiosperms [37,39,47-52]. Clearly, additional complete chloroplast genome sequences are needed to resolve the relationships among magnoliids, monocots, and eudicots.

A third incongruence between the MP and ML trees concerns the monophyly of the eurosid I clade (Figs. 2, 3). The MP tree (Fig. 2) strongly supports the monophyly of eurosid I (100% bootstrap), whereas in the ML tree the eurosid I clade in not monophyletic because *Cucumis *is strongly sister to the Myrtales instead of the Fabales. This same incongruence was detected in Jansen et al. [37] and was attributed to limited taxon sampling and model misspecification in ML analyses, two phenomena that are known to have adverse effects on phylogenetic reconstruction [53-57]. Expanded taxon sampling of rosids is needed to critically evaluate the monophyly of the eurosid I clade, especially since there is only moderate support for monophyly of eurosid I in previous phylogenies based on a single or few genes [reviewed in 58].

Both MP and ML trees are congruent with regard to the phylogenetic placement of *Citrus*. The genus is positioned as a member of the eurosid II clade, which has very strong bootstrap support in both MP (98%) and ML (100%) trees (Fig. 2). The eurosid II clade, which currently includes the four groups Brassicales, Malvales, Sapindales, and Tapisciaceae, has received strong support in previous DNA sequence phylogenies based on one to three genes [38], although relationships among these groups remain unresolved. Previous phylogenies based on whole chloroplast genomes [36,37,39,59] have included only one or two groups (*Arabidopsis*, Brassicales and/or *Gossypium*, Malvales). The addition of *Citrus *from the Sapindales expands the sampling to three of four currently recognized groups of eurosids II. Both MP and ML trees (Figs. 2, 3) provide strong support (98 – 100% bootstrap) for a sister relationship between the Brassicales and Malvales. This same relationship was weakly supported based on phylogenies using one or two chloroplast genes [46,60]. In contrast, the three gene phylogeny of Soltis et al. [38] weakly supported a sister relationship between the Malvales and Sapindales. Although taxon sampling is still somewhat limited, our 61-gene phylogeny provides very strong support for a close relationship between the Brassicales and Malvales. Expanded taxon sampling of the eurosid II clade is needed to confirm these results.

## Conclusion

Complete chloroplast genome sequences provide valuable information on spacer regions for integration of transgenes at optimal sites via homologous recombination, as well as endogenous regulatory sequences for optimal expression of transgenes and should help in extending this technology to other useful crops. Availability of complete chloroplast genome sequence should pave the way for genetic manipulation of *Citrus *and other members of the Rutaceae. Furthermore, the addition of the *Citrus *genome sequence to phylogenetic analyses provides strong support for the monophyly of the eurosid II clade, and the sister group relationship between the Sapindales and the Brassicales/Malvales clade.

## Methods

### Source of DNA

*Citrus sinensis *(L.) Osbeck var 'Ridge Pineapple' leaf tissue was chosen as the source plant material because it is being used in the US and international effort to sequence the *Citrus *genome. The lamellar tissue used was obtained from field-grown mature trees. Chloroplast DNA was isolated as described Jansen et al. [61]. Chloroplast DNA was subjected to rolling circle amplification (RCA) using the Repli-g kit following the manufacturers instructions (Molecular Staging Inc, New Haven, CT.).

### DNA sequencing and genome assembly

Purified RCA products were subjected to nebulization, followed by end repair and size-fractionated by agarose gel electrophoresis to obtain fragment lengths ranging from 2.0–3.5 kb. Repaired products were blunt-end cloned into pCR^®^-4Blunt-TOPO and then transformed into ElectroMax™ DH5alpha cells by electroporation (TOPO^® ^shotgun cloning kit, Invitrogen, Carlsbad, CA). Transformed cells were selected on LB agar containing 100 μg/μL ampicillin and arrayed into 30 × 96-well microtitre plates. Sequencing reactions were carried out in both the forward and reverse direction using the BigDye^® ^Terminator v3.1 Cycle sequencing kit and separated by a 3730xL DNA sequence analyzer (Applied Biosystems, Foster City, CA). Sequence data were assembled using Sequencher v4.5 (GeneCodes Ann Arbor, MI) following quality and vector trimming. Gap regions were filled by sequencing PCR fragments generated from primers designed to flank the gaps. The assembly was considered complete when sequence with confidence scores of ≥ 20 as judged by KB Basecaller software (Applied Biosystems) was accumulated at every base position with at least 4X coverage.

### Confirmation of IR expansion

To confirm the IR expansion that results in duplication of the genes *rps19 *and *rpl22*, PCR amplicons were generated that overlapped the junction of IRa and IRb with the LSC region. Primer sequences were as follows: *rpl22*F 5'-CAAAGCCCGCCAGGTAATTG-3' and *psbA*R 5'-CATTTCTTCCTGGCTGCTTG-3' for the amplicon overlapping IRa and LSC region and *rpl22*R 5'-GGAGAATTTGCGCCCACTAT-3' and *rps*F 5'-CTATCCGTGCAATTCCCTCA-3' for the amplicon overlapping IRb and LSC region. Following PCR, the amplicons were cloned into the pCR^®^4-TOPO vector following the manufacturer's instructions (Invitrogen), then sequenced using methods described above.

### Gene annotation

The *Citrus sinensis *genome was annotated using DOGMA [Dual Organellar GenoMe Annotator, 62]. Further, searches against a custom database of the previously published chloroplast genomic sequences using BLASTX were used to identify additional putative protein-coding genes. Both tRNAs and rRNAs were identified by searches against the same database using BLASTN.

### Repeat analysis

To determine the repeat structure of the *Citrus *chloroplast genome, REPuter [63] was used to identify the number and location of direct and inverted (palindromic) repeats using a minimum repeat size of 30 bp and a Hamming distance of 3 (i.e., repfind -f -p -l 30 -h 3 -best 10000).

### Variation between coding sequences and cDNAs

Positional determination of potential RNA edits was accomplished using 1505 cp sequences from GenBank without chromatographic traces in addition to in-house *Citrus sinensis *ESTs that contained chromatograms [64]. Only regions having a redundancy of at least four ESTs at each position were considered in the analysis. Differences were counted only when a base change was observed in the consensus sequence based on plurality. All assembly comparisons were made with the help of Sequencher v4.5.

### Phylogenetic analysis

Phylogenetic analysis was performed by using PAUP* version 4.10 b10 [65]. Phylogenetic analyses excluded gap regions to avoid ambiguity in regions where alignment was problematic. All MP searches included 100 random addition replicates and TBR branch swapping with the Multrees option. Modeltest 3.7 [66] was used to determine the most appropriate model of DNA sequence evolution for the combined 61-gene dataset. Hierarchical likelihood ratio tests and the Akaike information criterion were used to assess which of the 56 models best fit the data, which was determined to be GTR + G + I by both criteria. For ML analyses we performed an initial parsimony search with 100 random addition sequence replicates and TBR branch swapping, which resulted in a single tree. Model parameters were optimized onto the parsimony tree. We fixed these parameters and performed a ML analysis with three random addition sequence replicates and TBR branch swapping. The resulting ML tree was used to re-optimize model parameters, which then were fixed for another ML search with three random addition sequence replicates and TBR branch swapping. This successive approximation procedure [67] was repeated until the same tree topology and model parameters were recovered in multiple, consecutive iterations. Successive approximation has been shown to perform as well as full-optimization for both empirical and simulated datasets [67]. Non-parametric bootstrap analyses [68] were performed for MP analyses with 1000 replicates with TBR branch swapping, 1 random addition replicate, and the Multrees option and for ML analyses with 100 replicates with NNI branch swapping, 1 random addition replicate, and the Multrees option.

## Abbreviations

cpDNA, chloroplast DNA; IR, inverted repeat; SSC, small single copy; LSC, large single copy; bp, base pair; MP, maximum parsimony; ML, maximum likelihood; EST, expressed sequence tags; cDNA, complementary DNA; PCR, Polymerase Chain Reaction.

## Authors' contributions

MGB compared DNA and EST sequences for RNA editing, DNA sequencing and initial genome assembly and writing sections of the manuscript including those regarding RNA editing; NDS performed the repeat analyses, drew the circular map and assisted in writing the first and subsequent drafts of this manuscript; SBL isolated chloroplasts, performed RCA amplification of cpDNA, genome annotation, analysis and submission of data to the GenBank; RKJ assisted with extracting and aligning DNA sequences, performed phylogenetic analyses, and wrote the phylogenetic portions of this manuscript; HD conceived and designed this study, interpreted data, wrote several sections and revised several versions of this manuscript. All authors have read and approved the final manuscript.

## Figures and Tables

**Table 1:** Repeated sequences in the *Citrus sinensis *chloroplast genome.

Repeat Number	Size(bp)	Repeat	Location
1	30	I	IGS (*trnS-GCU, trnS-GGA*)
2	30	I	IGS (*rpl33 – rps18*)
3	30	D	IGS (*rrn5 – rrn4.5*)
4	31	I	IGS (*petN – psbM*)
5	31	D	IGS (*trnG-GCC – trnR-UCU*, *rpl32 – trnL-UAG*)
6	31	D	IGS (*trnsG-GCC – trnR-UCU*, *rpl32 – trnL-UAG*)
7	31	D	IGS (*trnF-GAA – ndhJ*, *rps12_3end – trnV-GAC*)
8	31	I	IGS (*trnF-GAA – ndhJ*, *trnV-GAC – rps12_3end*)
9	32	D	IGS (*trnG-GCC – trnR-UCU*)
10	33	I	IGS (*atpF – atpH*)
11	34	I	IGS (*psbZ – trnG-GCC*)
12	34	I	IGS (*trnS-GCU – trnG-GCC, psbM – trnD-GUC*)
13	34	D	IGS (*rrn4.5 – rrn5*)
14	34	I	IGS (*rrn4.5 – rrn5*)
15	34	I	IGS (*rrn4.5 – rrn5*)
16	34	D	IGS (*rrn5 – rrn4.5*)
17	36	I	IGS (*rpoB – trnC-GCA*)
18	36	I	IGS (*rpl32 – trnL-UAG*)
19	36	I	Intron (*ndhA*), IGS (*trnV-GAC – rps12_3end*)
20	36	D	IGS (*rps12_3end – trnV-GAC*), Intron (*ndhA – ndhA*)
21	38	I	IGS (*ycf4 – cemA*)
22	40	I	IGS (*psbT – psbN*)
23	41	D	IGS (*rps12_3end – trnV-GAC*), Intron (*ndhA – ndhA*)
24	41	I	Intron (*ndhA – ndhA*), IGS (*trnV-GAC – rps12_3end*)
25	41	D	*psaB*, *psaA*
26	44	D	*psbB, psaA*
27	48	I	IGS (*petN – psbM*)
28	51	I	IGS (*trnG-GCC – trnR-UCU*)
29	53	I	IGS (*trnS-GCU – trnG-GCC*, *psbM – trnD-GUC*)

The table includes the number and location of the repeats ≥ 30 bp, with a sequence identity greater than or equal to 90% (i.e., Hamming distance of 3). I-Inverted, D-direct, IGS-Intergenic spacer region.

**Table 2:** Comparison of the sweet orange chloroplast genome with EST sequences obtained from GenBank and in-house database of *Citrus sinensis *source.

Gene	Gene size (bp)	Sequence analyzed^**a**^	Number of variable sites	Variation type^**b**^	Position(s)^**c**^	Amino Acid change	Amino acid characteristics^**d**^
*petL*	93	1–93	1	C-U	5	P-L	HPONP-HPONP
*rps18*	303	1–303	1	C-U	227	I-I	HPONP-HPONP
*psbH*	219	1–219	1	C-U	137	V-A	HPONP-HPONP
*ycf2*	6840	1667–1947	2	C-A	5045	A-D	HPONP-HPIA
		5001–5841		A-U	5633	G-L	HPIP-HPONP
*ndhA*	1089	1–1089	1	C-U	344	S-L	HPIP-HPONP
*rrn23*	2810	1–2810	5	T-C	950	--	
				T-C	878	--	
				A-U	1196	--	
				A-G	1376	--	
				T-C	1706	--	

Putative RNA editing sites were determined by comparing EST sequence information from GenBank and the *Citrus *chloroplast genome sequence using Sequencher v 4.5. ^**a **^Gene sequence which considers the first base of the initiating codon as 1. ^**b**^Variation type: nucleotide in genomic DNA-nucleotide in mRNA. ^**c**^Variable position is referenced to the first base of the initiating codon of the gene sequence. ^**d**^HPONP-hydprophobic non-polar, HPIA-hydrophilic acidic, HPIP-hydrophilic polar.
